# Design and Implementation of Streaming Media Server Cluster Based on FFMpeg

**DOI:** 10.1155/2015/963083

**Published:** 2015-02-03

**Authors:** Hong Zhao, Chun-long Zhou, Bao-zhao Jin

**Affiliations:** School of Computer and Communication, Lanzhou University of Technology, Lanzhou 730050, China

## Abstract

Poor performance and network congestion are commonly observed in the streaming media single server system. This paper proposes a scheme to construct a streaming media server cluster system based on FFMpeg. In this scheme, different users are distributed to different servers according to their locations and the balance among servers is maintained by the dynamic load-balancing algorithm based on active feedback. Furthermore, a service redirection algorithm is proposed to improve the transmission efficiency of streaming media data. The experiment results show that the server cluster system has significantly alleviated the network congestion and improved the performance in comparison with the single server system.

## 1. Introduction

With the rapid development of streaming media technology and the expansion of its application fields, more and more users want to enjoy high definition video. As a result, large volume of media data needs to be transmitted over the network and the server loads increase dramatically [1]. In order to handle the massive volume of data, the streaming media system has to alleviate the load of backbone network to avoid network congestion.

In conventional streaming media single server system, all the tasks are processed by only one server. This kind of data processing causes low parallel service and poor capacity of streaming media server. It is difficult to meet the current network requirements of large quantity of VOD (video on demand) and low data transmission of the streaming media system. The streaming media server cluster with superb scalability and fault tolerance is an effective method to resolve the problem of servers overloading and provide high quality service. For the purpose of improving the service performance, tasks are assigned to different nodes by using load-balancing scheduling mechanisms [2–4]. With regard to streaming media system, most of the studies focus on the load-balancing algorithm. For instance, Wonjae Lee propose a load-balancing system for IPTV Web application virtualization which supports high availability and distributes tasks based on server loads [5]. A new load-balancing policy, named ADuS, is proposed in [6], which attempts to partition jobs according to their present sizes and further rank servers based on their loads. Although these algorithms have been used to improve the performance of streaming media server cluster in the simulation experiments, they did not consider the whole layout and specific applications. And few of them have proved their feasibilities in a real streaming media server cluster system.

However, there are only a few studies focusing on the protocols. For example, in [7], the streaming media content type and streaming protocols are used to relieve the bandwidth bottleneck and make smooth transmission of streaming media data. But when the number of the users increases, the service performance will become unsatisfactory. FFMpeg [8] is the excellent software to provide streaming media system platform. It not only contains some crucial and indispensable tools for streaming media system, such as FFPlay, FFServer [9, 10], but supports most of the codec which can enhance the applicability of streaming media system, such as H.264, MPEG [11, 12].

In this paper, we propose a scheme to construct a streaming media server cluster based on FFMpeg. In this server cluster, the user management of partition is used to scatter streaming media data in various areas, which can alleviate the load of backbone network and avoid network congestion. Meanwhile, a dynamic load-balancing algorithm based on active feedback and an improved redirection algorithm are used to improve the service ability of the streaming media system.

## 2. The Limit of Streaming Media Single Server System Based on FFMpeg

In the streaming media single server system based on FFMpeg, all relevant manipulations, such as authentication, establishing connection, and providing streaming media data, are processed by FFServer. However, with the increasing of VOD quantity, the bandwidth and server loads will be increased, which becomes the bottleneck of streaming media single server system based on FFMpeg. The analysis of occupied bandwidth is described as follows:

(1) $$\begin{array}{c} W_{s}=\overset{K}{\underset{i=1}{\sum }}N_{i}, \end{array}$$

where *N*_*i*_ is the occupied bandwidth of user *i*  (*i* = 1,2,…, *k*) and *W*_*s*_ is the output bandwidth of FFServer. From (1), the larger the *K*, the greater *W*_*s*_. It indicates that server loads and backbone bandwidth of the network occupied by server will be increased by the number of connected users. All of these do not adapt to the development of the streaming media technology.

## 3. Design of Streaming Media Server Cluster System Based on FFMpeg

**Figure 1:**
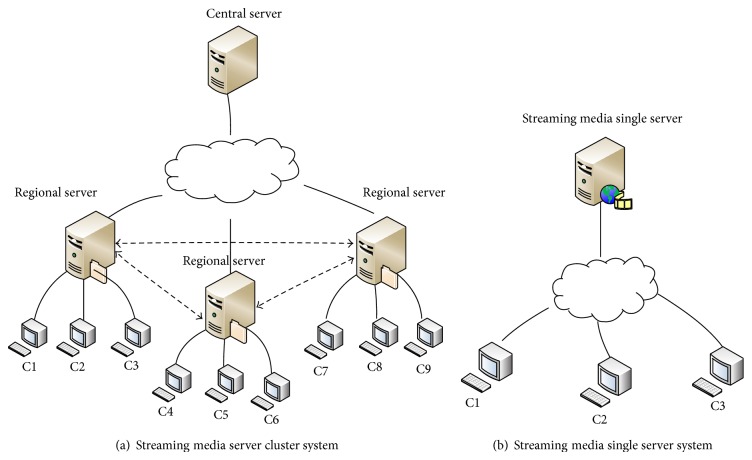
Two kinds of streaming media service system models.

Compared with streaming media single server system, the streaming media server cluster system based on FFMpeg consists of a central server and multiple regional servers that are deployed on the edge of the network with multiple copies of objects. The load-balancing and redirection algorithms are used by the central server to select optimal servers for users so that the data transmission is in relatively small scope. Thus, the pressure on the backbone network can be alleviated effectively. Two kinds of streaming media system models are shown in Figure 1.

### 3.1. Design of Server Cluster

#### 3.1.1. Design of Central Server

*Central Server*. The central server, which is the center of the streaming service system, is implemented based on FFServer. For the purpose of better serving the VOD requests which are sent by users, the central server not only has massive storage and powerful data processing ability but also holds copies of all the media files, all the regional servers' IP and the streaming media server cluster diagrams. By using a specific scheduling algorithm, it keeps the server loads in balance. Meanwhile, it can provide streaming data to other users and regional servers. Some indispensable elements are implemented as follows.

**Figure 2:**
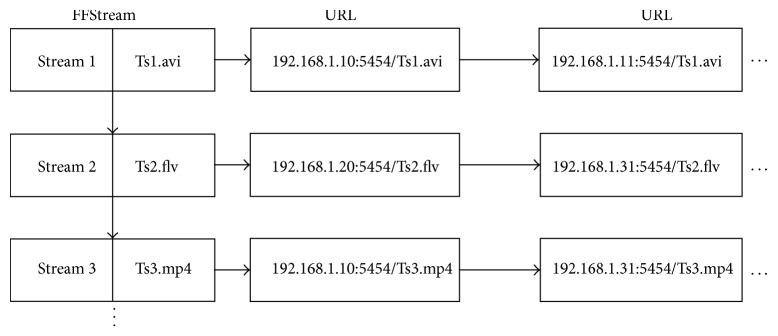
Video streaming list.

*(1) The Establishment of the Video Streaming List.* In the central server, a crucial data structure, named FFStream, records all the information of each multimedia stream. All the media files are stored in first_stream that is a list linked to FFStream. Furthermore, as shown in Figure 2, the URL (uniform resource locator) is the full path to other servers which include corresponding streaming media files.

**Figure 3:**
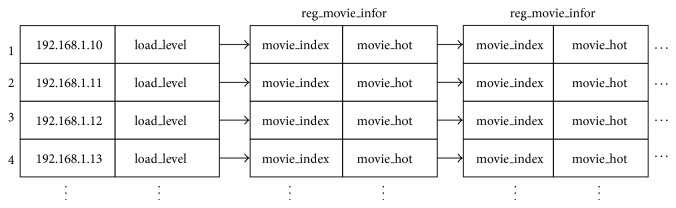
Server addresses array.

*(2) The Establishment of Server Addresses Array.* The array is used to store all servers' IP address and load conditions of each server (load_level) and the information of regional movie (reg_movie_infor). As shown in Figure 3, the information of regional film (reg_movie_infor) is defined as follows:

 Struct reg_movie_infor
 
{
 Int movie_index;
 Int movie_hot;
 };

movie_index: the positions of requested movie (it has not been downloaded to regional server) in the first_stream.

movie_hot: demand-rate (demand times per day) of the movie (no more than the specific threshold).

**Figure 4:**
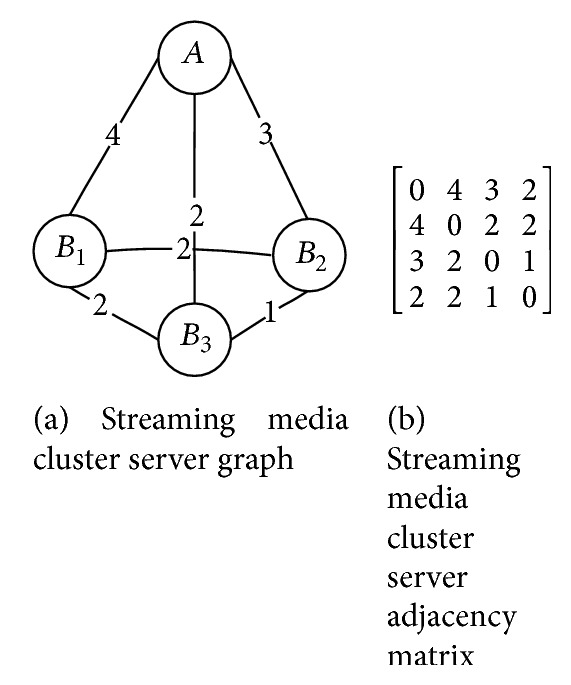
Cluster service graph and adjacency matrix.

*(3) The Establishment of Streaming Media Server Cluster Graph.* It is a weighted undirected graph which is composed by the routing hops among servers and stored in an adjacency matrix. The weight of each edge represents the corresponding routing hops between two servers. By using the adjacency matrix and video streaming list, the central server selects the optimal server which has a relatively small cost and smooth route for the user to transmit streaming data. An example is shown in Figure 4, where *A* represents the central server, and *B*_*i*_  (*i* = 1,2 …, *n*) represents the regional server.

#### 3.1.2. Design of Regional Server

*Regional Server*. There are multiple regional servers in the streaming media service system, and all of them are deployed in different regions, which are only responsible for specific region transmission of the media data and only store the higher-demand movies. Compared to the central server, the regional servers have lower demands for storage but require powerful processing capacity because they are responsible for data transmission among the whole regions. Regional servers can be implemented in using FFServer. They play dual rules: one is the server that provides streaming data for the user when it receives requirement of VOD and the other is the user that will download the media resources from other servers.

### 3.2. The Schedule Process of Server Cluster System

**Figure 5:**
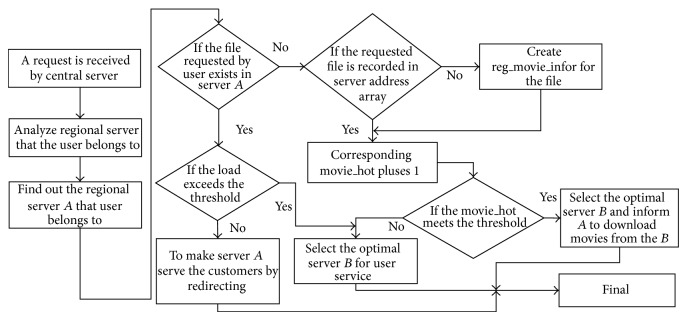
Schedule process of system.

The central server controls the schedule process of the server cluster system. The schedule process is shown in Figure 5.

### 3.3. The Analysis of Network Bandwidth

For Figure 3, assume that *K* users are distributed to *T* regional servers and *N*_*i*_ is the occupied bandwidth of network when user *i* (*i* = 1,2, 3,…, *K*) demands media resources. The total bandwidth of all the users is *W*_*s*_ = ∑_*i*=1_^*K*^ *N*_*i*_. The average bandwidth is

(2) $$\begin{array}{c} \bar{N}=\frac{\sum_{i=1}^{K}N_{i}}{K}. \end{array}$$

According to YU et al. analysis of China telecom Power Info system data shows that approximate demand of 80% is concentrated on the film of 23%, which follows the 80–20 rule. Based on this, 20% of the hot films are stored in regional servers in this system, if *K* users are distributed to *T* regional servers on average, (1–80%) of K users need to get streaming data from other servers instead of their own regional servers; meanwhile, some regional servers need to download requested data from other servers. Therefore, the occupied bandwidth of backbone is defined as follows:

(3) $$\begin{array}{c} W_{c}=20\%K\bar{N}+D=20\%\overset{K}{\underset{i=1}{\sum }}N_{i}+D, \end{array}$$

where *D* is the total bandwidth occupied by regional servers that download the streaming media files from other servers, which is given by

(4) $$\begin{array}{c} D=\frac{20\%Kd}{H}, \end{array}$$

where *H* is the heat threshold of regional films and *d* is the bandwidth of downloading video of a single region server. Representing *d* using the average bandwidth, the occupied bandwidth of backbone can be rewritten as follows:

(5) $$\begin{array}{c} W_{c}=20\%\overset{K}{\underset{i=1}{\sum }}N_{i}+\frac{20\%K\times t\bar{N}}{H}. \end{array}$$

The parameter *t* can be set dynamically according to the network congestion degree and server performance. When *t* < 4 · *H*, we can get inequalities (6) from formula (5). Consider the following:

(6) $$\begin{array}{c} W_{c}=\left(\frac{20\%t}{H}+20\%\right)\cdot W_{s}<W_{s}. \end{array}$$

Obviously, inequality (6) shows that the streaming media server cluster can reduce the bandwidth of backbone network compared with the streaming media single server system.

## 4. Scheduled Algorithm of Server Cluster System

### 4.1. Load-Balancing Algorithm

Load-balancing is to effectively disperse a large volume data to a limited number of regional servers, which provides load balance between servers and avoids network bottleneck.

In the existing load-balancing algorithms [13, 14], the dynamic load-balancing algorithm is well-developed, such as the content-based dynamic load-balancing algorithm [15]. However, the algorithms do not use real-time feedbacks. In this paper, a dynamic load-balancing algorithm using active feedback is proposed and described as follows.

(1) Regional servers monitor their own load situation in real time by getting their own load information *I*_*s*_, which is defined as follows:
  (7) $$\begin{array}{c} I_{s}=\sqrt{\frac{I_{C}^{2}+I_{D}^{2}+I_{M}^{2}}{3}}, \end{array}$$
 where *I*_*C*_ is occupation rate of CPU, *I*_*M*_ is occupation rate of memory, and *I*_*D*_ is occupation rate of hard disk. Meanwhile, every regional server will set the threshold values *B*_*M*_, *B*_*C*_, and *B*_*D*_ for each of their own conditions of hardware loads *I*_*M*_, *I*_*C*_, and *I*_*D*_. And the field of 10*I*_*s*_ is divided into five grades, that is, grade_1_, grade_2_, grade_3_, grade_4_, and threshold grade_*s*_, which meets the following condition: 0 < grade_*i*_ < grade_*j*_ < grade_*s*_ < 10  (0 < *i* < *j* < 5), where grade_*s*_ is set based on the actual situation. The divided process of grade_*i*_ is shown as follows:
  (8)  grade1=0+grades2, grade3=grade1+grades2, grade2=grade1+grade32, grade4=grade3+grades2.
(2) When regional servers find that their own load grade has changed, they will send the information of changed grade, that is, the feedback of local load grade_*i*_, to the central server immediately. If the hardware loads of the regional servers or whole loads reach the threshold value, the regional servers will send the corresponding threshold value *B*_*t*_  (*t* = *M*, *C*, *D*) or grade_*s*_.
(3) When the central server receives the information of changed loads level, it will update the corresponding regional server loads grade in the server addresses array immediately for routing selection. If it receives the loads grade which is the threshold value, the central server will not assign tasks for the regional servers.
(4) When the central server selects the optimal server *S*_*j*_ for user *i*, it looks up the server cluster adjacency matrix, finds out the route hops from regional server *S*_*i*_ to *S*_*j*_, and retrieves server loads grade from the server addresses array. The optimal server *S*_*j*_ which is selected gives the maximum value of (9):
  (9) $$\begin{array}{c} \text{MAX}\left\{\left.\frac{1}{1/\text{grad}{\text{e}}_{j}+N_{ij}}\right\}\right.\;\left(j=1,2,\ldots ,n,\;\;i\neq j\right), \end{array}$$
 where grade_*j*_ is the load grade of *S*_*j*_ and *N*_*ij*_ is the route hops from regional server *S*_*i*_ to the optimal server *S*_*j*_.

### 4.2. Redirection Algorithm

In conventional redirection algorithms, user requests are sent to the central server which selects the optimal server and returns its IP back to the user. Then the user will resend the request to the optimal server to obtain streaming data. There is a drawback in this redirection algorithm that the user must wait until the center server returns the optimal server IP. As a result, the response time is long.

In this paper, the redirection algorithm is modified to reduce the response time. The algorithm is described as follows.

1. When the requests are sent to the central server, the central server will select an optimal server for the user who sends the request and transmit the request to the optimal server. Meanwhile, the optimal server's IP will be sent to the user.
2. When the optimal server receives the request which is transmitted from the central server, it establishes connection for the user immediately and sends central server IP and confirmation information to the user. Then it provides streaming data to the user directly.
3. When the user receives the returning information from central server or optimal server, it will get ready to receive streaming data.

## 5. Analysis of Experiment

Hardware platform: CPU: E3-1230 V2, 3.3 GHz. Memory: 4 G, DDR3 1600. Ethernet: 100 M.

Software platform: OS: Ubuntu 12.04 STL. Virtual machine: VMware Workstation 9.0. Virtual machine disk: 20 G. Virtual machine memory: 2 G. Bandwidth test tool: Bmon, Luyougang. On-demand software: FFPlay. Test move: 4508 kbps.

Streaming media single server system: FFServer is used as the server.

Streaming media server cluster is based on FFMpeg: One central server and two regional servers have been set in this experiment. Users have been distributed across two regional servers on average. The heat threshold *H* is set to 2. The downloading bandwidth of a single server is 4508 kbps. The loads_level of each server is grade_1_ = 4, grade_2_ = 5, grade_3_ = 6, and grade_4_ = 7 and the threshold value grade_*s*_ is 8. During the experiment, the demanded streaming media files do not exist in their own server with 20% probability, and Bmon and Luyougang tools are used to detect network bandwidth at the same time. All the recorded data are illustrated in Table 1.

The data given in Table 1 shows that when the number of users increases up to 20 in the single server system, the bandwidth of backbone network increases to 11.32 by degrees. Until the number of users reaches 22, the bandwidth is close to the upper limit load of Ethernet card equipped in the single server. As a result, the response time becomes significantly longer and the packages begin to be lost which lead to an unsmooth video. However, in the server cluster system, the load and bandwidth of the central server have declined significantly. The two regional servers undertake most of data transmission, which can guarantee loads balance of the system basically. Due to the fact that all the data are scattered in various areas effectively, bandwidth of backbone network is much smaller than the single server system.

## 6. Conclusion

In this paper, we proposed an advanced load-balancing algorithm and an improved redirection algorithm for the streaming media server cluster based on FFMpeg. The system not only provides load-balancing among servers but also reduces the bandwidth of backbone network effectively. In the future work, the technology of voluntary server and P2P will be introduced to the streaming media cluster system to establish the video grid of multilevel scalable architecture.

## Figures and Tables

**Table 1:** The comparison of bandwidth occupied by each of servers (unit: MB/s).

Total number of users	Single server (bandwidth of FFServer)	Server cluster								
		Backbone bandwidth	Central server		Regional server 1			Regional server 2		
			Foreign users	Bandwidth	Foreign users	Local users	Bandwidth	Foreign users	Local users	Bandwidth
10	5.73	2.20	2	1.96	0	5	2.22	0	5	2.26
12	6.87	0.80	0	0.42	1	4	2.88	0	8	4.10
14	7.98	1.56	2	1.49	0	6	2.90	0	8	4.23
16	9.13	1.88	1	0.77	1	6	3.41	1	10	5.12
18	10.64	2.20	1	0.89	2	8	5.04	0	10	4.51
20	11.32	3.19	4	2.94	0	10	4.51	0	10	4.49
22	—	2.63	1	1.23	1	16	7.88	2	6	4.01
24	—	2.66	2	2.03	1	16	7.80	1	8	4.66
26	—	3.41	3	3.22	1	16	7.88	1	10	5.10
28	—	2.88	2	2.26	1	16	7.85	2	12	6.88
